# In vivo generated autologous plasmin assisted vitrectomy in young patients

**DOI:** 10.1186/s40942-022-00391-3

**Published:** 2022-06-11

**Authors:** Cengiz Aras, Fevzi Senturk, Arif Koytak, Mahmut Dogramaci, Sevil Karaman Erdur, Selim Kocabora

**Affiliations:** 1 Department of Ophthalmology, Istanbul Medipol Medical School, Istanbul, Turkey; 2 Department of Ophthalmology, Medical Faculty, Bezmialem Vakif University, Istanbul, Turkey; 3 MBchB, MD, ICO, MRCOphth The Princess Alexandra Hospital NHS Trust, London, UK

**Keywords:** Posterior vitreous detachment, Tissue plasminogen activator, Autologous whole blood

## Abstract

**Background:**

Autologous plasmin enzyme facilitates the induction of posterior vitreous detachment(PVD) during vitrectomy in young patients. We proposed the concept of in-vivo generated plasmin which is based on the injection of tissue plasminogen activator(t-PA) and autologous whole blood(AWB) into the vitreous cavity. The purpose of this pilot study is to report the efficacy of preoperative simultaneous intravitreal injection of(t-PA) and autologous whole blood in facilitating the intraoperative induction of PVD in young patients with various vitreoretinal pathologies.

**Methods:**

Seventeen eyes of 16 young patients with various vitreoretinal pathologies requiring vitrectomy, who received simultaneous intravitreal injection of 0.1 ml of AWB and 25 µg of t-PA, 3 days prior to surgery were retrospectively reviewed. Outcome measures were the number of attempts required to achieve successful intraoperative separation of the posterior hyaloid; the postoperative visual acuity; and intraoperative and postoperative complications.

**Results:**

The mean age of the patients was 23.87 ± 10.09 years, ranging from 10 to 39 years. Eight of 16 patients were men. The mean follow-up time was 19.35 ± 5.04 months, ranging from 12 to 26 months. Surgical indications for vitrectomy were chronic retinal detachment (n = 7), traumatic retinal detachment without proliferative vitreoretinopathy(n = 3), traumatic macular hole(n = 1), secondary vasoproliferative tumor(n = 4) and optic pit maculopathy(n = 2). Patients with retinal detachment complicated with PVR and those who were older than 40 years of age were excluded from the study. Separation of the Weiss ring from the optic nerve head was achieved intraoperatively in all cases, with a mean number of 2.86 ± 1.4 attempts. While the mean preoperative LogMAR visual acuity was 1.38 ± 0.59, ranging from 2.40 to 0.50, it was a mean of 0.51 ± 0.29, ranging from 1.00 to 0.10 at final postoperative exam(p < 0.001; paired samples t-test). No preoperative or intraoperative complications were noted.

**Conclusion:**

Preoperative simultaneous intravitreal injection of 25 µg t-PA with 0.1 ml of AWB facilitates the intraoperative induction of posterior vitreous detachment in young patients.

## Background

Surgical induction of posterior vitreous detachment (PVD) is an important step of vitreoretinal surgery in patients with no preexisting posterior vitreous detachment. Failure to create a complete posterior vitreous detachment may jeopardize the overall outcome of the procedure. In the adult age group, PVD can be induced by active aspiration of the vitreous gel from the nasal side of the optic nerve head [1]. However, despite improvements in vitreoretinal instruments and techniques, the surgical induction of PVD in pediatric and young patients remains challenging. Enzymatic vitreolysis is a potential way of inducing PVD, particularly in pediatric and young vitreoretinal patients [2].

Enzymatic vitreolysis has been studied for the last thirty years. Among many potential agents, plasmin is the most promising. It has been reported that intravitreal injection of plasmin in animal studies and autologous plasmin enzyme(APE) in human studies facilitates PVD induction during vitrectomy [3–6].

The currently available techniques of purifying autologous plasmin are complex and expensive. The main components of plasmin generation are plasminogen and t-PA. We proposed the concept of in vivo generated autologous plasmin, which is based on the existence of the main components of plasmin generation in the same media. We used t-PA and autologous whole blood as a source of plasminogen.

In this pilot study, we investigated the efficacy of simultaneous preoperative intravitreal injection of t-PA and autologous whole blood in young patients with no preexisting PVD who required vitrectomy for various vitreoretinal pathologies, to facilitate PVD induction during surgery.

## Materials and methods

The study was performed in a retrospective, nonrandomized and uncontrolled manner. The charts of 17 eyes of 16 young patients who had no preexisting PVD, and received intravitreal injection of 25 µg t-PA with autologous whole blood, 3 days prior to vitrectomy for various vitreoretinal pathologies were reviewed. Procedures were performed between June 2018 and September 2020 at Istanbul Medipol University Hospital, Turkey, a tertiary referral center. Patients who had retinal detachment complicated with proliferative vitreoretinopathy, had no clear vitreous cavity, or were older than 40 years age were excluded from the study. All patients received complete ophthalmological examination, including visual acuity with a Snellen Chart, slit-lamp biomicroscopy, intraocular pressure measurement, and indirect biomicroscopic fundoscopy with a wide angle fundus contact lens. Color fundus photography and optical coherence tomography taken at the macula and optic nerve head were performed preoperatively in all cases. Age, sex, pre- and postoperative visual acuity, lens status, vitreoretinal pathology, surgical techniques, complications and time of the last follow-up exam were obtained from notes. Visual acuity scores were converted into LogMAR.

This study was approved by the Medical Ethical Committee of Istanbul Medipol University and was conducted in accordance with the tenets of the Declaration of Helsinki. Written- informed consent from the patients and/or guardians was obtained before the procedures. Patients were prepared in the usual manner for any intravitreal injection, including 3 min of 5% povidone iodine application, draping, and lid speculum insertion. After anterior chamber paracentesis of 0.1–0.2 ml aqueous humor, 0.1 ml autologous whole blood taken from the brachial vein, was immediately injected into the vitreous cavity in the superior quadrant, 3 mm behind the limbus using a 27 G needle under topical anesthesia. Twenty-five micrograms of t-PA(Actylise, Boehringer Ingelheim, Ingelheim am Rhein, Germany) in 0.05 ml was also injected intravitreally with a 30 G needle. Topical antibiotics were given for three days after injection; 23-gauge pars plana vitrectomy under general anesthesia was performed 3 days after the intravitreal injections.The induction of posterior vitreous separation was attempted after core vitrectomy by suctioning the vitreous at the nasal side of the optic disc with a vacuum pressure of 400 mmHg (The Constellation Vision System platform, Alcon,Fort Worth, TX, USA). The number of attempts to achieve separation of the posterior hyaloid was noted. A PVD occurred if a Weiss ring was freely separated from the retina. In some cases, vitrectomy was combined with extracapsular lens extraction by phacoemulsification, with or without intraocular lens implantation. Long-acting gas or silicone oil were used as tamponading agents.

## Results

**Fig. 1:**
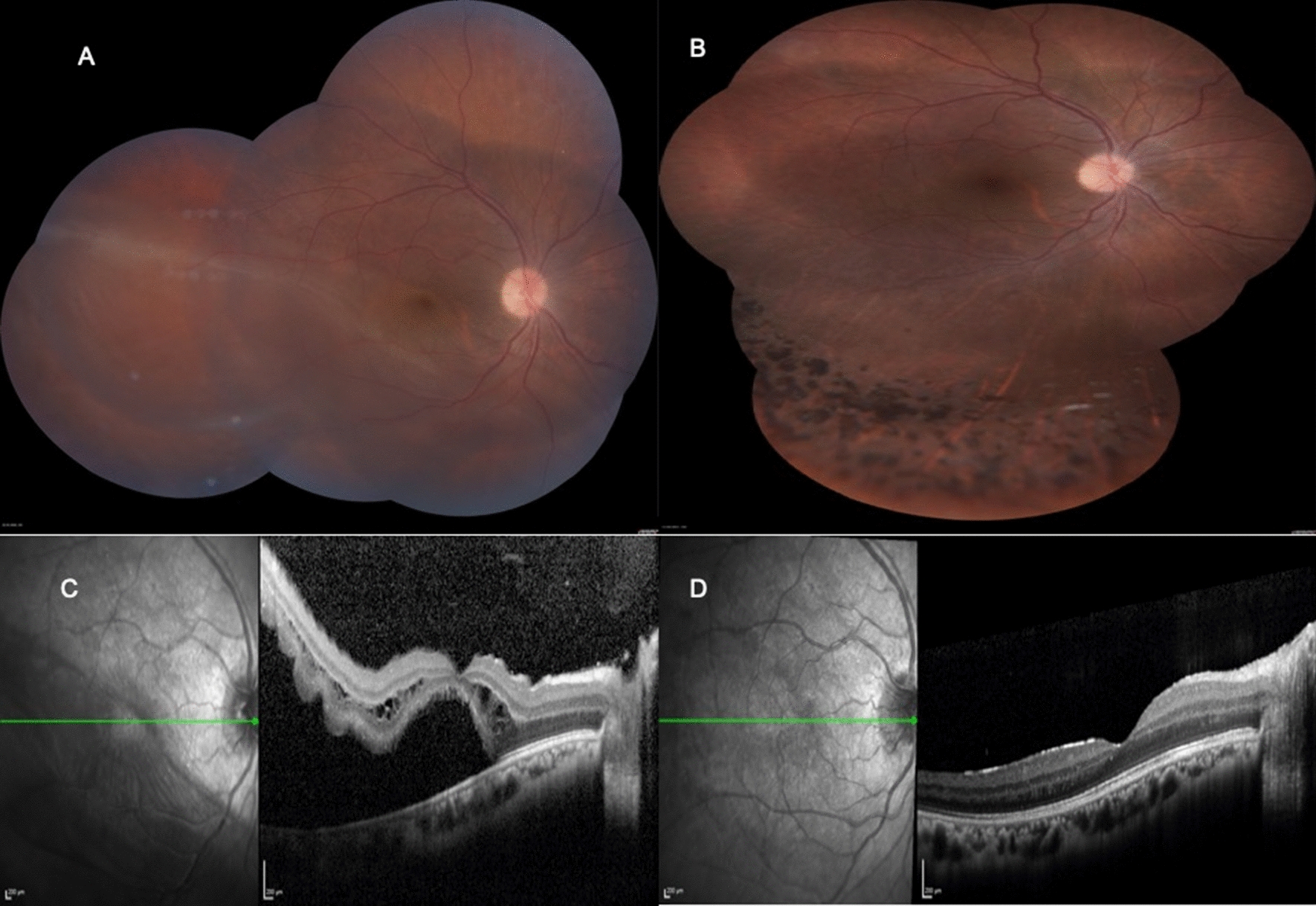
Pre (**A**) and postoperative (**B**) color fundus photographs and postoperative OCT (**D**) of a patient with chronic retinal detachment. Note that there is no posterior vitreous detachment preoperatively (**C**)

**Fig. 2:**
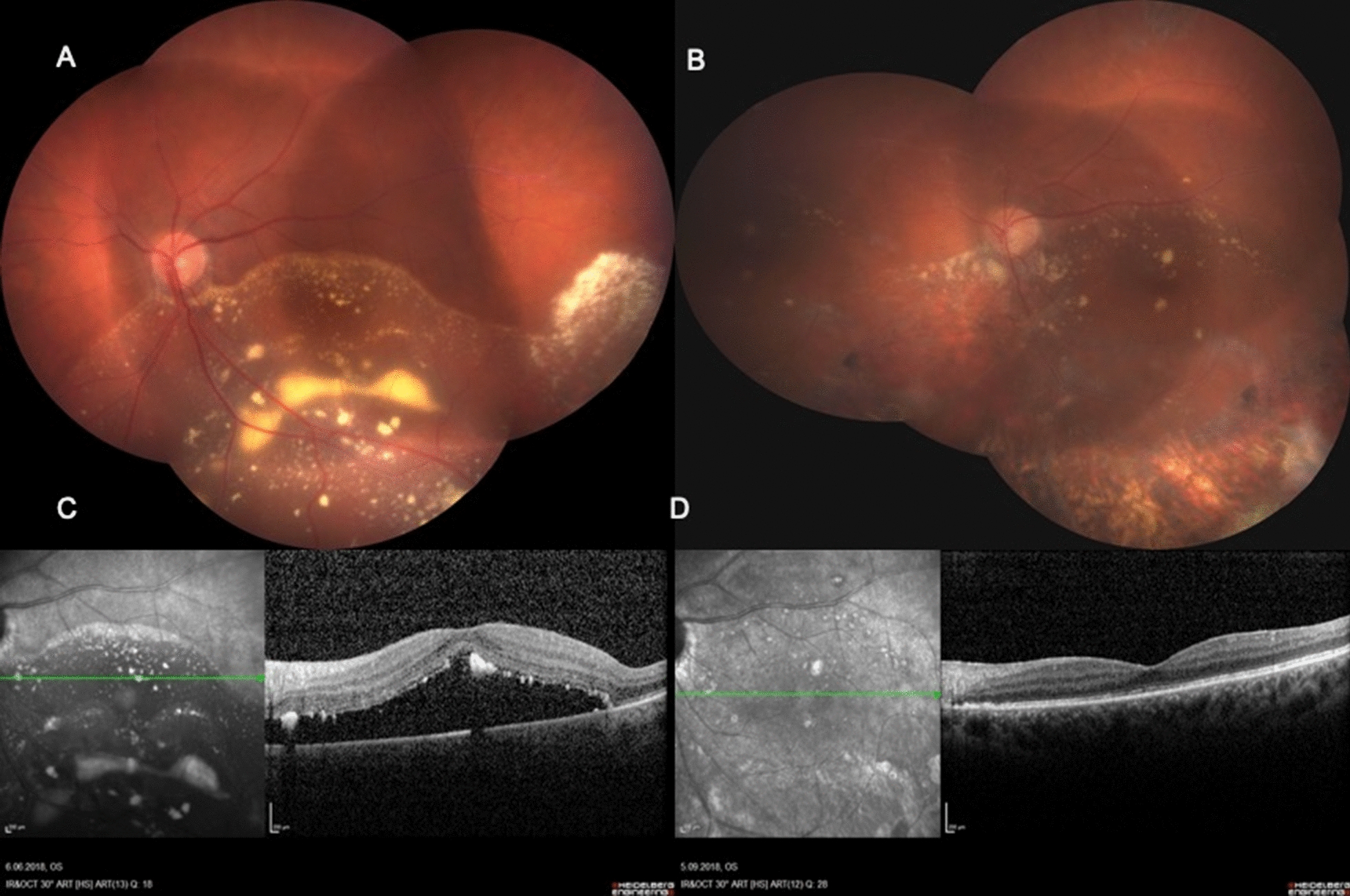
Color fundus photographs and OCT of a patient with a vasoproliferative tumor, taken pre (**A**, **C**) and postoperatively (**B**, **D**). Note that the retina is completely attached with a normalized macular contour (**D**)

The mean age of the patients was 23.87 ± 10.09 years, ranging from 10 to 39 years. Eight of 16 patients were men. One patient had bilateral surgery. Patient characteristics are shown in Table 1. All surgeries were performed by a single, experienced surgeon (CA). Surgical indications for vitrectomy were chronic retinal detachment with subretinal membrane formation (n = 7) (Fig. 1), traumatic retinal detachment without proliferative vitreoretinopathy (n = 3), traumatic macular hole (n = 1), secondary vasoproliferative tumor (n = 4) (Fig. 2) and optic pit maculopathy (n = 2). Three of 7 patients with chronic retinal detachment had a past history of failed buckling surgery. Four eyes were already pseudophakic. All patients underwent vitrectomy. In 8 of 17 eyes, vitrectomy was combined with extracapsular lens extraction by phacoemulsification, with or without intraocular lens implantation and silicone oil injection. PVD induction during vitrectomy was achieved in all cases. The mean number of attempts for separation of the Weiss ring from the optic nerve head was 2.86, ranging from a minimum of 1 to a maximum of 4. Postoperative tamponading agents were silicone oil in 13 eyes, and sulfure hexafluoride gas in 4 eyes. Silicone oil removal and simultaneous intraocular lens implantation surgeries in aphakic cases were performed 8–12 weeks after surgery.

**Table 1:** Patient characteristics

Age	Gender/eye	Diagnosis	Inj-Op ınterval (day)	Treatment	No of attempts for PVD	Preop VA LogMAR	Postop VA LogMAR	Final status	Follow-up time (month)	Complication
39	M/L	Chronic RD	3	PPV, LE	3	2.00	0.70	Attached	23	None
12	F/L	Chronic RD	3	PPV	2	2.40	0.70	Attached	18	Re-RD
31	F/R	Chronic RD	3	PPV	3	2.40	1.00	Attached	14	ERM
17	M/R	Traumatic RD	3	PPV, LE	1	1.00	0.70	Attached	14	None
13	M/L	Traumatic RD	3	PPV, LE	3	0.70	0.20	Attached	26	None
12	F/L	OPM	3	PPV	4	1.30	0.30	Attached	14	None
39	M/R	Traumatic RD	3	PPV	1	1.80	0.70	Attached	15	None
39	F/L	VPT	3	PPV	2	1.30	0.10	Attached	26	None
10	M/R	MH	3	PPV	2	1.00	0.70	Attached	25	None
19	M/R	VPT	3	PPV, LE	2	2.00	0.40	Attached	23	None
29	F/L	Chronic RD	3	PPV	1	1.30	0.10	Attached	12	None
23	F/R	Chronic RD	3	PPV, LE	2	1.80	0.50	Attached	18	None
21	M/L	VPT	3	PPV, LE	2	1.00	1.00	Attached	25	Re-RD
21	F/L	OPM	3	PPV	2	1.30	0.70	Attached	14	None
19	M/L	VPT	3	PPV, LE	2	0.50	0.50	Attached	23	ERM, Re-RD
24	F/L	Chronic RD	3	PPV, LE	1	1.30	0.40	Attached	23	None
33	M/R	Chronic RD	3	PPV, LE	1	0.50	0.10	Attached	16	None

PPV, pars plana vitrectomy; Inj-Op, ınjection-operation; PVD, Posterior vitreous detachment; VA, visual acuity; RD, retinal detachment; SiO, silicone oil; LE, lens extraction; MH, macular hole; OPM, optic pit maculopathy; VPT, vasoproliferative tumor

The mean follow-up time was 19.35 ± 5.04 months, ranging from12 to 26 months. Visual acuity improved in all cases. While the preoperative LogMAR visual acuity was 1.38 ± 0.59, ranging from 2.40 to 0.5, the mean LogMAR visual acuity was 0.51 ± 0.29, ranging from 1.00 to 0.10 at the final postoperative exam (p < 0.001; paired samples t-test).

No complications such as ghost cell glaucoma, endophthalmitis or unexpected reactions occurred related to the simultaneous injection of autologous whole blood and t-PA. Retinal detachment in 3 eyes and an epiretinal membrane in 2 eyes developed during the postoperative period. Among patients who developed retinal detachment after surgery, the previous diagnoses were vasoproliferative tumors in 2 cases and chronic retinal detachment in one case. All patients underwent a redo operation, and their retinas remained attached.

## Discussion

PVD induction during surgery is one of the main surgical steps in vitreoretinal procedures. In contrast to that in adult patients, the induction of PVD in young and pediatric age groups remains challenging.

Among many potential agents for enzymatic vitreolysis, plasmin is the most promising. Plasmin, which is a nonspecific serine protease, induces dose-dependent and complete PVD without causing any ultrastructural changes in the retina [7]. Autologous plasmin enzyme, t-PA and autologous serum have been used in the past to facilitate PVD by liquefying the vitreous and disrupting the vitreoretinal interface [7–11]. Chuang et al. Showed that preoperative injection of t-PA with autologous serum in eyes that were scheduled to undergo vitrectomy was effective for PVD induction in pediatric age groups [9]. In that study, they injected only t-PA into eyes that already had intravitreal blood. The authors proposed that the t-PA would catalyze the conversion of plasminogen, which was present in the autologous serum and intravitreal blood into plasmin in the vitreous cavity. In contrast, ocriplasmin, which is a recombinant protease enzyme, was not found to be effective for inducing PVD in pediatric age group [11].

The currently available techniques of purifying autologous plasmin are complex and expensive. We proposed the concept of invivo-generated autologous plasmin, which is based on supplementation of plasminogen and t-PA into the vitreous cavity. We used autologous whole blood as a source of plasminogen and intravitreally injected t-PA and autologous whole blood to generate autologous plasmin enzyme in the vitreous cavity of young patients, who were scheduled to undergo vitrectomy for various vitreoretinal pathologies. Three days after the injections, the patients underwent vitrectomy in which posterior vitreous detachment was easily achieved during surgery. We combined autologous whole blood with t-PA to catalyze the conversion of plasminogen in whole blood into plasmin. Achievement of posterior hyaloid separation in these young patients, whom the posterior hyaloid is expected to be firmly adherent to retina, increased the final success of surgical intervention in our case series.

A more pronounced proteolytic effect requires a delay of several hours between injection and vitrectomy. When t-PA is injected intravitreally, it takes 2–5 h until the highest concentrations of vitreal plasmin are generated from plasminogen [10]. Clinically, posterior vitreous detachment was induced in 7 of 10 patients with proliferative diabetic retinopathy 3 days after injection of t-PA [12]. We waited for three days after injection, as we supposed this would allow the plasmin to bind more substrate at the vitreoretinal surface and produce more efficacy.

We chose to use fresh autologous whole blood instead of autologous serum or plasma, as fresh autologous whole blood is a nontoxic and readily available source of plasminogen. It might also contain higher levels of plasminogen when compared to stored autologous serum or plasma, as plasminogen is an unstable product and its level declines rapidly under in vitro conditions [13]. On the other hand, the existence of fibrinogen in whole blood facilitates the activation of t-PA [14]. We observed no complications related to the simultaneous injection of autologous whole blood and t-PA.

Patients with proliferative vitreoretinopathy were excluded from the study because of the possible risk of rapid deterioration of the proliferative vitreoretinopathy as a result of the existing higher levels of growth factors and plasminogen activator inhibitors in the vitreous of such patients [15].

The limitations of our study are its retrospective nature, absence of a control group and relative subjectivity of the method of achieving PVD induction. However, the performance of surgeries by a single experienced surgeon decreased the possible bias related to subjectivity.

## Conclusions

Preoperative simultaneous intravitreal injection of 25 µg t-PA with 0.1 ml of AWB facilitates the intraoperative induction of posterior vitreous detachment in young patients.

For validation of this technique in a more authenticated manner, it needs to be studied further in a controlled, larger series.

## Data Availability

Not applicable.

## Abbreviations

PVD: Posterior vitreous detachment
t-PA: Tissue plasminogen activator
AWB: Autologous whole blood
APE: Autologous plasmin enzyme
